# Door-to-Balloon Time Outperforms ST-Segment Elevation in Predicting the STEMI vs. NSTEMI Final Diagnosis

**DOI:** 10.3390/jcm14186588

**Published:** 2025-09-18

**Authors:** José Nunes de Alencar, Harvey Pendell Meyers, William Frick, Jesse T. T. McLaren, Stephen W. Smith

**Affiliations:** 1 Instituto Dante Pazzanese de Cardiologia, Research Division, São Paulo 04012-909, Brazil; jose.alencar@dantepazzanese.org.br; 2 Department of Emergency Medicine, Carolinas Medical Center, Charlotte, NC 28203, USA; pendellmeyers@gmail.com; 3 Division of Cardiology, SSM Saint Louis University Hospital, St. Louis, MO 63104, USA; 4 Emergency Department, University Health Network, Toronto, ON M5G 2C, Canada; jesse.mclaren@gmail.com; 5 Department of Emergency Medicine, University of Minnesota Hennepin Healthcare, Minneapolis, MN 55455, USA

**Keywords:** acute myocardial infarction, ST-elevation myocardial infarction (STEMI), non-ST-elevation myocardial infarction (NSTEMI), occlusion myocardial infarction (OMI), percutaneous coronary intervention (PCI)

## Abstract

**Background:** The STEMI/NSTEMI classification guides management and quality metrics for acute myocardial infarction (AMI). We examined whether the final cath-lab diagnosis of STEMI versus NSTEMI correlates more closely with door-to-balloon (D2B) time than with either ST-segment elevation (STE) on pre-angiogram ECG or a culprit lesion with TIMI 0-1 flow. **Methods:** This retrospective study analyzed 410 patients with AMI from the DOMI-ARIGATO database who underwent coronary angiography. For each patient, we recorded FDx coded by the interventional cardiologist, D2B < 120 min versus > 120 min, STE criteria (Fourth Universal Definition), and angiographic TIMI 0-1 culprit. Predictors of FDx-STE discordance were evaluated with multivariable logistic regression. **Results:** Among 410 angiographed AMI patients (mean age 63 ± 13; 71% male), 165 (40.2%) received an FDx-STEMI and 245 (59.8%) an FDx-NSTEMI. D2B time showed 94% agreement with FDx (160/165 FDx-STEMI treated < 120 min; 225/245 FDx-NSTEMI treated > 120 min), exceeding concordance for STE (82%; *p* < 0.001) and TIMI 0-1 flow (75%; *p* < 0.001). FDx and STE diverged in 75 patients (18%): 60 rapidly treated STE-negative cases were labelled STEMI, whereas 15 delayed STE-positive cases were labelled NSTEMI. In regression analysis, D2B < 120 min remained the sole independent predictor of discordance (adjusted OR 6.7, 95% CI 3.5–13.8). **Conclusions:** In this registry, the cath-lab label “STEMI” showed the strongest correlation with meeting a 120 min benchmark, exceeding correlations for STE or angiographic occlusion. These findings suggest that quality-metric compliance, rather than electrocardiographic or anatomic criteria, predominantly drives final diagnosis.

## 1. Introduction

Among patients with acute myocardial infarction (AMI), the diagnosis of ST-elevation myocardial infarction (STEMI) versus non-ST-elevation myocardial infarction (NSTEMI) is formally defined by the Fourth Universal Definition of AMI [1]. This definition serves as the foundation for guideline recommendations distinguishing emergent from non-emergent reperfusion therapy, as well as for AMI-related quality assurance metrics and accreditation standards. However, the STEMI criteria frequently fail to align with the actual presence of acute coronary occlusion myocardial infarction (ACOMI, or OMI), which would plausibly benefit from emergent reperfusion therapies [2]. This misalignment has led many researchers to advocate replacing the STEMI/NSTEMI paradigm with one that reflects the underlying pathophysiology rather than a singular feature (ST elevation) of a single diagnostic test (the ECG). This alternative framework has been termed the OMI/Non-OMI paradigm, which has been increasingly discussed in recent literature [3,4].

While many physicians claim to adhere strictly to STEMI criteria when diagnosing STEMI and NSTEMI, there is evidence suggesting that, in practice, this adherence may be flexible. This raises a critical question: is “STEMI” defined strictly by “diagnostic ST elevation,” or does it also encompass “its equivalent”? We therefore prespecified one primary question: does achieving a door-to-balloon (D2B) time < 120 min show stronger agreement with the final interventional-cardiologist diagnosis (FDx-STEMI vs. FDx-NSTEMI) than either (a) the presence of guideline-defined ST-segment elevation on any pre-angiogram ECG or (b) angiographic evidence of an acute culprit lesion with TIMI 0-1 flow? The present analysis, anchored in a prospectively maintained two-center registry, was designed to test this hypothesis and to quantify the relative contribution of each marker to diagnostic discordance.

## 2. Methods

This study is a subanalysis of the DOMI-ARIGATO registry, a dataset that was purpose-built to study occlusion MI and therefore combines three prospectively captured, consecutive streams of suspected ACS patients from the same two hospitals [5,6]. First, both centers exported every urgent or emergent cardiac-catheterization activation (“code-STEMI” and other cath-lab callbacks) over a 12-month period, yielding a high-risk cohort with detailed timing fields and angiograms. Second, to avoid over-representing activation pathways, Stony Brook independently logged every patient admitted to the cardiology service with a working ED diagnosis of possible ACS during the subsequent six months; these admissions include many non-activation NSTEMI presentations. Third, to ensure an adequate control pool of abnormal ECGs without true occlusion, we merged a consecutive sample of Hennepin patients from the UTROPIA study whose ED tracings showed STE, ST depression, or marked T-wave inversion but who ultimately proved to have NOMI [7].

Patients were included if the IC explicitly documented a final diagnosis of either STEMI or NSTEMI in the cardiac catheterization report. Cases were excluded if no explicit final diagnosis was provided or if there was “late presentation AMI.” Late presentation was defined as instances where the IC documented that the patient presented beyond the time window in which emergent invasive management would have been indicated, typically due to prolonged symptom onset or delayed medical contact.

For each patient included, we extracted several key data points, including the final diagnosis (FDx-STEMI or FDx-NSTEMI) as determined by the operating IC, the presence or absence of STEMI criteria on all pre-angiogram ECGs (STEc+ or STEc−), the location of the culprit lesion, the TIMI flow grade, the peak troponin value, and the time interval from hospital presentation to coronary angiography. Additional clinical and procedural characteristics relevant to the study objectives were also collected.

To standardize definitions, an ECG was classified as STEc+ if it met the STEMI criteria as per the Fourth Universal Definition of Myocardial Infarction, which define STE as new ST-segment elevation at the J-point in two or more contiguous leads with the following cutoffs:

- ≥2.5 mm in men < 40 years in leads V2 and V3;
- ≥2.0 mm in men ≥ 40 years in leads V2 and V3;
- ≥1.5 mm in women (all ages) in leads V2 and V3;
- ≥1.0 mm in other contiguous leads (non-V2/V3).

The presence of STEMI criteria was determined by blinded review of every pre-angiography ECG by two independent readers—an experienced emergency physician (SWS) and a board-certified cardiologist. We adopted a conservative rule: a case was classified as STE-positive if either reader identified guideline-defined ST-segment elevation. A patient was considered positive for STEMI criteria if any pre-angiogram ECG met these formal criteria. For cases where STEMI criteria were absent on the initial ECG but evolved on subsequent pre-angiogram ECGs, the time from the first STEMI-positive ECG to angiography was used as the relevant time interval rather than the time from hospital presentation.

OMI was defined as the presence of an acute culprit lesion with TIMI 0 or 1 flow on coronary angiography. This assessment was based on the findings documented in the cardiac catheterization report by the operating IC. In the present analysis, therefore, OMI functions as an angiographic outcome variable that categorizes cases according to confirmed vessel-level obstruction, independent of clinical presentation or ECG findings. The final diagnosis recorded in the catheterization report was classified as FDX-STEMI or FDX-NSTEMI, irrespective of the presence or absence of STEc+. TIMI flow grade was recorded as reported, with TIMI 0 indicating no flow and TIMI 1 indicating minimal perfusion beyond the site of occlusion.

Timely treatment was defined as that occurring within 120 min from hospital presentation to angiography. Although STEMI guidelines specify a door-to-device time of ≤90 min for primary PCI, a slightly more inclusive threshold of <120 min was chosen for this study because some patients in this cohort were transferred from an initial medical facility to one of the two participating PCI centers, where the guideline-recommended target for door-to-device time is <120 min in cases involving inter-hospital transfer. This threshold also aligns with NSTEMI guidelines, which recommend intervention within two hours for patients with ongoing ischemic symptoms, making it a pragmatic cutoff to capture aggressive care in both populations.

To ensure accuracy and replicability, all cardiac catheterization reports in the database included the full text written by the operating IC, and these reports were independently reviewed by one of the authors (HPM). This blinded review process confirmed the FDx of STEMI or NSTEMI as recorded by the IC, incorporating all known angiographic findings and clinical events at the time of diagnosis.

### Statistical Analysis

All data were managed in Microsoft Excel (Microsoft Corporation, Redmond, WA, USA) and analyzed using GraphPad Prism version 10.1.0 (GraphPad Software, LLC, San Diego, CA, USA). Descriptive statistics were used to summarize baseline characteristics and clinical outcomes of the study population. To compare the agreement between paired categorical variables assessed within the same patient population, such as the concordance between final diagnosis (dichotomized), presence or absence of STEMI criteria, time to treatment (categorized as greater or less than 120 min), and TIMI flow grade (categorized as 0–1 or 2–3), McNemar’s test was performed. Predictors of diagnostic discordance (STE criteria ≠ FDx) were assessed with multivariable logistic regression. Both univariate and multivariate logistic regression analyses were conducted. Univariate regression was performed to identify variables significantly associated with discordance, and variables with a 95% confidence interval (CI) that excluded the null value (OR = 1) were included in the multivariate model to account for potential confounders. To assess collinearity, variance inflation factors (VIFs) were calculated. Additionally, we applied the Hosmer–Lemeshow test to evaluate the model’s calibration. For these collinearity assessments, R version 4.4.2 was used (R Foundation for Statistical Computing, Vienna, Austria). Statistical significance was defined by 95% confidence intervals that did not include the null value. Conceptually, we posit a simple hypothesis in which (i) clinical findings such as ST-segment elevation and angiographic occlusion may prompt rapid reperfusion and (ii) the resulting door-to-balloon interval, now fixed in the chart, can in turn influence the cath-lab label. Under this structure, treatment time lies on the causal pathway between physiology and the administrative diagnosis, so including STE itself as a covariate would induce collider bias; hence, STE was analyzed only in pair-wise comparisons and not entered the multivariable model.

We also present a Sankey flow diagram. Its construction followed the open-source networkD3 library in R; nodes represent the four dichotomous variables (STE±, door-to-balloon ±120 min, TIMI 0-1 yes/no, and FDx), and link widths are proportional to patient counts.

## 3. Results

**Figure 1:**
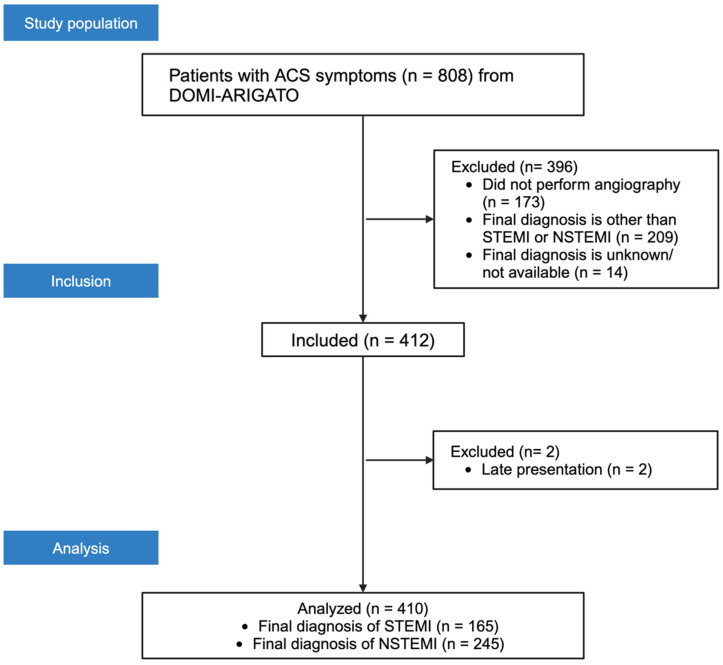
Legend: Inclusion, exclusion, and analysis flow diagram of patients from the DOMI-ARGATO database.

From 808 registry entries, 635 patients underwent coronary angiography; 225 had an explicit cath-lab diagnosis other than AMI and were excluded, leaving 410 analyzable encounters (Figure 1). Within this cohort, 165/410 (40.2%) were assigned a final diagnosis of STEMI and 245/410 (59.8%) a final diagnosis of NSTEMI. Baseline demographics and clinical features are summarized in Table 1.

When we compared each marker with the final diagnosis, a D2B time < 120 min showed the highest agreement: 160/165 STEMI cases and 225/245 NSTEMI cases were concordant, yielding 385/410 (94%) overall agreement. Guideline ST-segment-elevation criteria were concordant in 105/165 STEMI and 230/245 NSTEMI patients—335/410 (82%; McNemar *p* < 0.001 vs. D2B). Agreement fell further for an angiographic culprit with TIMI 0-1 flow: 112/165 STEMI and 191/245 NSTEMI patients were concordant, 303/410 (74%; McNemar *p* < 0.001 vs. D2B).

In the subgroup where the final diagnosis did not match the STE criteria (*n* = 75; 18%), the final diagnosis was aligned with time to treatment in 71 cases. Most discordant cases (60 of 75) involved patients who were diagnosed with FDX-STEMI despite being STEc−, a pattern strongly associated with rapid time to treatment (<120 min). The other 15 patients were diagnosed as FDX-NSTEMI despite being STEc+.

**Figure 2:**
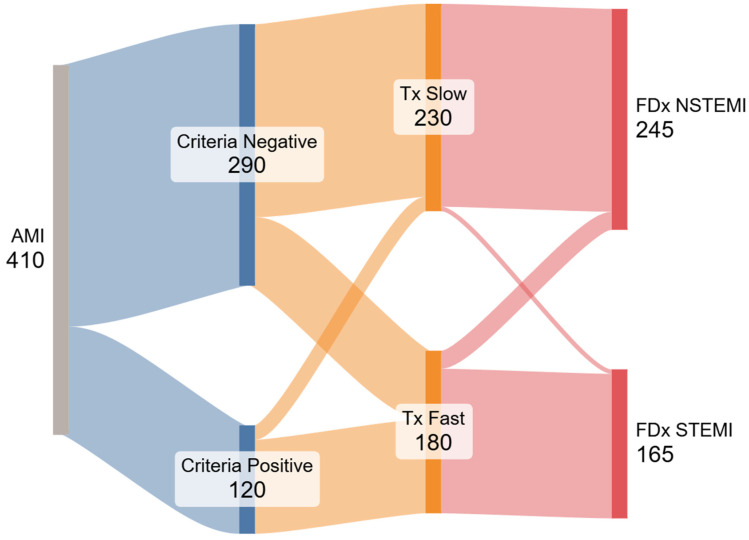
Legend: Sankey diagram illustrating the discrepancies between STEMI criteria, time to treatment, and FDx of STEMI or NSTEMI. The diagram highlights the divergence from the expected STEMI/NSTEMI paradigm, showing how time to treatment and final diagnosis often misalign with initial ECG findings.

The occurrence of divergent diagnoses—defined as a final diagnosis inconsistent with the presence or absence of STE criteria—was significantly more frequent among patients treated within <120 min (60 of 180 rapid treatment; 33.3%) compared to those treated after >120 min (15 of 230 cases; 6.5%) (*p* < 0.0001). Among the 15 cases misclassified as FDx-NSTEMI despite being STEc+, 13 underwent angiography after >120 min. Conversely, among the 60 cases misclassified as FDx-STEMI while being STEc−, 58 underwent angiography within <120 min. We generated a Sankey diagram (Figure 2) based on our results to visually illustrate the divergence between STEMI criteria, treatment time, and final diagnosis.

In this model, STE could not be an independent variable because it is embedded in the outcome definition (divergent diagnosis). Regarding the prespecified logistic regression analysis, multiple clinical variables were evaluated to identify factors associated with disagreement in the FDx. A univariate logistic regression was initially performed to assess individual predictors of disagreement versus agreement in FDx. Variables found to be significantly associated with disagreement included time to treatment < 120 min, presence of TIMI 0-1 flow, stent placement, and heart failure at admission. Subsequently, a multivariate logistic regression was conducted including only these significant variables to adjust for potential confounders. In the multivariate model, time to treatment < 120 min remained the only statistically significant predictor of diagnostic disagreement (OR: 6.7, 95% CI: 3.45–13.82), while TIMI 0-1, stent placement, and heart failure at admission were no longer significant. These results are summarized in Table 2.

We evaluated the risk of overfitting by considering the events per variable (EPV) ratio in the multivariate model. With 18.75 events per variable, the analysis was statistically well-supported. Additionally, the Hosmer–Lemeshow test was conducted to assess calibration, yielding a non-significant result (*p* = 0.91) and variance inflation factors (VIFs) were all close to 1, suggesting minimal collinearity.

## 4. Discussion

**Figure 3:**
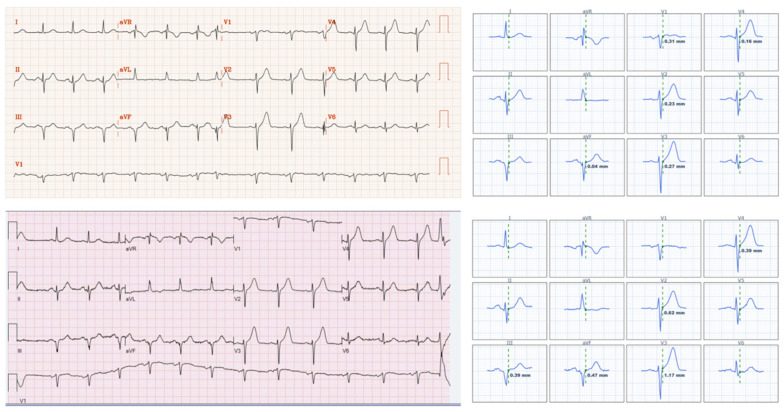
Legend: An adult presenting with acute chest pain, who had an angiogram performed 83 min after arrival, with acute culprit lesions in the mid-LAD (TIMI 2 flow, 90% stenosis) and distal LAD (TIMI 0 flow, 100% stenosis) each requiring PCI. Final interventional cardiologist diagnosis is “STEMI.” Only two ECGs were performed before the angiogram, both shown above, without STEMI criteria, but with hyperacute T waves in the anterolateral distribution.

**Figure 4:**
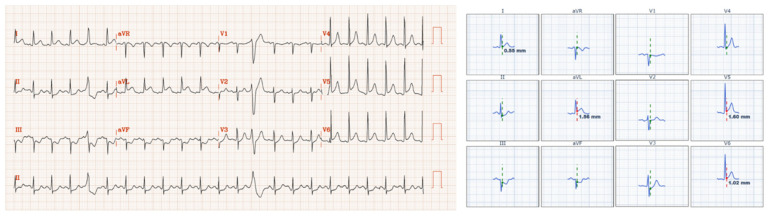
Legend: Adult patient presenting with acute chest pain; angiogram done at 1942 min (32.4 h) after arrival. First ECG on arrival shown above. Five ECGs were performed in the 32 h from arrival to angiogram, with the first three ECGs positive for STEMI criteria starting at 23:33 at night through 07:54 the next morning. Later that afternoon, repeat ECGs no longer met STEMI criteria. Angiogram at 32 h showed culprit second diagonal culprit thrombotic occlusion, but some collateral noted to backfill the vessel at that time. In the setting of triple vessel disease, the patient underwent CABG within the hospitalization. Final diagnosis was NSTEMI.

Our multivariable analysis asked a specific question: what factors explain the 18% of encounters in which the cath-lab label contradicts the patient’s ECG? Overall, door-to-balloon time < 120 min was far more concordant with the final cath-lab diagnosis than either guideline ST-segment elevation or angiographic TIMI 0-1 occlusion (94% vs. 82% vs. 74%). This observation answers our prespecified question and underscores that, in routine practice, the label applied in the cath report aligns most closely with the treatment timeline recorded in the electronic chart. This suggests that the designation of STEMI is not always a reflection of the original diagnostic framework, but rather a retrospective label influenced by procedural outcomes and time benchmarks. Figure 3 and Figure 4 illustrate these discrepancies.

A key observation was that cases with discordant final diagnoses and STE criteria followed a predictable pattern. The direction of discordance was highly patterned: 60 STE-negative but rapidly treated cases were coded as STEMI, whereas 15 STE-positive but delayed cases were coded as NSTEMI. Importantly, the cath-lab diagnosis is entered after angiography, once all timing fields are fixed in the electronic record; the label therefore cannot have influenced how quickly the team moved. One might expect the opposite sequence—guideline STEc would trigger rapid reperfusion and thus predict the time stamp—but our data show that, in practice, the time stamp more often predicts the label. Consistent with this, multivariable logistic regression confirmed that a D2B time < 120 min was the only independent predictor of STE/FDx disagreement, whereas angiographic occlusion and other clinical features lost significance.

One might argue that our finding is tautologic—that STE triggers fast PCI and therefore predicts the STEMI label. However, the discordance framework deliberately removes STE from the model; the persisting association of treatment speed with the opposite ECG category demonstrates that the time benchmark itself is influencing how the label is applied.

To further illustrate this phenomenon, we performed a simple interpretive comparison that treated each marker as if it were a diagnostic test for the final label. When viewed this way, a treatment time < 120 min “identified” STEMI with 97% sensitivity and 92% specificity (335 convergent diagnosis in 410 patients), whereas presence or absence of STEc identified it with only 64% sensitivity and 94% specificity (335 convergent diagnosis in 410 patients). To clarify, this analysis does not measure the accuracy of *STE criteria* or *time to treatment* in identifying acute coronary occlusion or the true presence of myocardial infarction—this was done in other articles by our team [3,8]. Existing evidence indicates that the paradigm greatly underestimates many cases of acute occlusion, with a sensitivity of STEMI criteria for OMI of only 43% [2].

These findings illustrate a potential limitation in the STEMI/NSTEMI paradigm: the final coding may fail to reliably capture the underlying pathology it was intended to represent. As the 2025 ACC/AHA/ACEP/NAEMSP/SCAI Guideline for the Management of Patients with Acute Coronary Syndromes claims, ‘Patients with NSTEMI may have a partially occluded coronary artery leading to subendocardial ischemia, while those with STEMI typically have a completely occluded vessel leading to transmural myocardial ischemia and infarction,’ accompanied by a figure showing NSTEMI with partially occlusive thrombus and STEMI with completely occlusive thrombus [9]. Instead, we found the final diagnosis of STEMI appears to be driven more by treatment timelines than by the presence of STE criteria or acute coronary occlusion. This observation raises concerns about the medical significance of the STEMI/NSTEMI classification, as it suggests a disconnection between the final diagnosis and the pathophysiological state of the myocardium. Rather than serving as a marker of acute coronary occlusion, the label of STEMI appears to be a surrogate for whether emergent reperfusion was performed. This pattern reflects the influence of quality measures and institutional benchmarks on clinical decision making. These findings underscore the limitations of the current STEMI/NSTEMI paradigm as both a clinical decision-making tool and a quality metric [8]. Prior studies have quantified the diagnostic accuracy of guideline STE for detecting angiographic occlusion [2]. By contrast, our study—to our knowledge the first to do so—tests the specific hypothesis that door-to-balloon time aligns more closely than STE or TIMI 0-1 with the final cath-lab label (STEMI vs. NSTEMI).

The Sankey diagram visually illustrates the multiple ways in which the STEMI classification is inconsistently applied in clinical practice and fails to reflect actual patient outcomes. Under the idealized STEMI/NSTEMI framework, two parallel pathways should emerge: patients meeting STEMI criteria would receive rapid reperfusion, have a higher likelihood of a completely or subtotally occluded coronary artery, and receive a discharge diagnosis of STEMI. In contrast, patients without STE would receive delayed angiography, more often present with open arteries on angiography, and be diagnosed as NSTEMI. However, our data revealed significant deviations from this theoretical model. Whether the same ‘time-drives-diagnosis’ pattern holds in community hospitals with different EMS throughput warrants prospective study, we believe this is a systemic issue.

This inconsistency may have important implications for both clinical practice and quality improvement metrics. The reliance on the STEMI/NSTEMI framework can obscure opportunities for care improvement. For instance, subtle occlusions treated rapidly were often labeled as STEMI, while patients with significant delays in intervention despite meeting STE criteria were categorized as NSTEMI. As a result, missed occlusions—cases of delayed intervention despite critical pathology—may be often concealed under the NSTEMI label rather than being highlighted as areas for improvement in systems of care. Conversely, successful identification of electrocardiographically subtle occlusions treated rapidly was not appropriately recognized as a positive deviation from the standard STEMI definition. This phenomenon aligns with the previously described “no false negative paradox”, where NSTEMI patients with acute myocardial infarction due to acute coronary occlusion are not classified as missed occlusions [8].

Our data reinforce the need for objective OMI-detection tools such as AI-ECG or prehospital scoring to decouple triage decisions from administrative time metrics.

## 5. Limitations

First, the analysis was conducted in only two high-volume PCI centers, which may limit the generalizability of the findings to other institutions with differing patient populations, resource availability, and clinical practices. While high-volume centers often represent best-case scenarios for timely intervention, they may not reflect care patterns in lower-volume or community hospitals where protocol adherence and access to rapid reperfusion may vary significantly. As this is a retrospective analysis of patients undergoing coronary angiography, there is an inherent selection bias, as lower-risk NSTEMI cases that did not warrant an invasive approach may not have been included. In addition, 225 angiographed cases in the source registry lacked an unequivocal final diagnosis in the dictated report and were excluded a priori. Chart review showed that most of these omissions reflected unstable angina labels, missing dictation templates, or late-presentation AMI; nevertheless, removing them may have introduced a second layer of selection bias that we cannot fully quantify. Because NSTEMI patients managed non-invasively were not captured, our findings apply only to cases in which a cath-lab diagnosis was rendered. However, it is the practice at both institutions to perform angiography on all patients with acute MI that is presumed to be Type 1 due to ACS.

TIMI grades were abstracted verbatim from operator reports without core-lab adjudication, introducing potential misclassification. Because TIMI 0-1 was used only as a comparator marker, any non-differential error would bias against our hypothesis.

Because interventionalists dictate the final diagnosis after reviewing angiographic findings and with full knowledge of the actual D2B interval, and perhaps of early clinical course, classification bias is possible. Such awareness, however, would amplify the observed association between treatment speed and the administrative label.

Finally, despite the rigorous methodology and inclusion of key variables such as ECG findings and angiographic data, this study is limited by the potential influence of unmeasured confounders. These variables were not directly captured in the regression model and could contribute to the observed association between time to treatment and the final diagnosis, raising the possibility of residual confounding.

## 6. Conclusions

In this retrospective analysis, time to treatment showed the strongest association. in final diagnosis of STEMI vs. NSTEMI, more important than both STEMI millimeter “criteria” and the presence or absence of total coronary occlusion. This has implications for research and quality improvement.

## Figures and Tables

**Table 1:** Baseline characteristics of patients with a final diagnosis of STEMI and NSTEMI.

	FDx-STEMI (N = 165)	FDx-NSTEMI (N = 245)
Stony Brook University Hospital	137 (83%)	183 (75%)
Hennepin County Medical Center	28 (17%)	62 (25%)
Average age (years)	61.34	66
Female	42 (25%)	75 (31%)
Caucasian	134 (81%)	190 (78%)
Hx of diabetes	41 (25%)	99 (40%)
CKD	7 (4%)	40 (16%)
CHF	7 (4%)	45 (18%)
Hx of HTN	104 (63%)	177 (72%)
Hx of tobacco use	99 (60%)	152 (62%)
Hx of known CAD	30 (18%)	117 (48%)
Chest pain as a presenting symptom	145 (88%)	171 (70%)
Dyspnea as a presenting symptom	70 (42%)	121 (49%)
Time to cath (mins), median (IQR)	35 (20–57)	1106 (259–2803)
Time to cath < 120 min	160 (96%)	20 (8%)
STEMI criteria met on ECG	105 (64%)	15 (6%)
ECG judged OMI+ by blinded expert	160 (97%)	91 (37%)
OMI outcome (original definition)	149 (90%)	101 (41%)
Culprit lesion	154 (93%)	177 (72%)
Stent placed	147 (89%)	137 (60%)
CABG performed during hospitalization	6 (4%)	14 (6%)
HCMC peak trop I (mg/dL)	N = 15, median 29.86, mean 85.27	N = 48, median 5.02, mean 25.45
SBUH peak trop T (mg/dL)	N = 126, median 3.78, mean 6.46	N = 157, median 0.290, mean 1.03
Mean LVEF	49%	49%
Presence of focal wall motion abnormality	137 (86%)	130 (61%)
Cardiac arrest during visit	22 (13%)	12 (5%)
Vasopressor use during hospitalization	24 (15%)	20 (8%)
Death during hospitalization	10 (6%)	7 (3%)

Legend: Abbreviations: CABG: coronary artery bypass graft, CAD: coronary artery disease, CHF: congestive heart failure, CKD: chronic kidney disease, ECG: electrocardiogram, FDx: final diagnosis, HCMC: Hennepin County Medical Center, HTN: hypertension, HX: history, IQR: interquartile range, LVEF: left ventricular ejection fraction, NSTEMI: non-ST-elevation myocardial infarction, OMI: occlusion myocardial infarction, SBUH: Stony Brook University Hospital, STEMI: ST-elevation myocardial infarction, TIMI: thrombolysis in myocardial infarction.

**Table 2:** Predictors of diagnostic discordance (outcome = final diagnosis ≠ guideline STE criteria).

Variable	Univariate OR (95% CI)	Multivariate OR (95% CI)
Hospital [Hennepin]	1.154 (0.6254 to 2.050)	–
<120 min	7.167 (3.998 to 13.60)	6.727 (3.451 to 13.82)
TIMI 0-1	2.166 (1.307 to 3.615)	0.7994 (0.4327 to 1.471)
Stent performed	2.708 (1.450 to 5.459)	1.281 (0.6141 to 2.794)
Night and weekend	0.6600 (0.2433 to 1.514)	–
Age (years)	0.9946 (0.9756 to 1.014)	–
Diabetes	0.7563 (0.4313 to 1.290)	–
DLP	1.076 (0.6467 to 1.815)	–
HTN	0.9053 (0.6917 to 2.107)	–
Sex	0.8818 (0.5160 to 1.543)	–
Prior MI	0.9053 (0.4524 to 1.702)	–
Prior PCI	0.6505 (0.3413 to 1.175)	–
Prior CABG	0.7958 (0.2913 to 1.848)	–
Congestive heart failure at admission	0.2432 (0.05798 to 0.6879)	0.4157 (0.09510 to 1.275)
Tobacco use	0.9393 (0.5653 to 1.578)	–
Chest pain	1.415 (0.7447 to 2.890)	–
Dyspnea	1.377 (0.8165 to 2.341)	–

Legend: Abbreviations: CABG: coronary artery bypass graft, DLP: dyslipidemia, FDx: final diagnosis, HTN: hypertension, MI: myocardial infarction, NSTEMI: non-ST-elevation myocardial infarction, PCI: percutaneous coronary intervention, TIMI: thrombolysis in myocardial infarction.

## Data Availability

Data are available upon request.
